# Genome-wide identification of MAXs genes for strigolactones synthesis/signaling in solanaceous plants and analysis of their potential functions in tobacco

**DOI:** 10.7717/peerj.14669

**Published:** 2023-01-12

**Authors:** Lixianqiu Wang, Bingjie Li, Changbo Dai, Anming Ding, Weifeng Wang, Haoqi Shi, Mengmeng Cui, Yuhe Sun, Jing Lv

**Affiliations:** 1 Key Laboratory for Tobacco Gene Resources, Tobacco Research Institute of Chinese Academy of Agricultural Sciences, Qingdao, China; 2 Graduate School of Chinese Academy of Agricultural Sciences (GSCAAS), Beijing, China

**Keywords:** Strigolactone biosynthesis, Strigolactone signal transduction, Cis-elements, *MAXs* gene family, Shoot development, Abiotic stress

## Abstract

The more axillary growth (*MAX*) gene family is a group of key genes involved in the synthesis and signal transduction of strigolactones (SLs) in plants. Although *MAX* genes play vital roles in plant growth and development, characterization of the *MAX* gene family has been limited in solanaceous crops, especially in tobacco. In this study, 74 members of the *MAX* family were identified in representative *Solanaceae* crops and classified into four groups. The physicochemical properties, gene structure, conserved protein structural domains, cis-acting elements, and expression patterns could be clearly distinguished between the biosynthetic and signal transduction subfamilies; furthermore, *MAX* genes in tobacco were found to be actively involved in the regulation of meristem development by responding to hormones. *MAX* genes involved in SL biosynthesis were more responsive to abiotic stresses than genes involved in SL signaling. Tobacco *MAX* genes may play an active role in stress resistance. The results of this study provide a basis for future in-depth analysis of the molecular mechanisms of *MAX* genes in tobacco meristem development and stress resistance.

## Background

Strigolactone (SL) is a novel phytohormone produced in plant roots. Studies have shown that SLs inhibit plant branching, shape root morphology, promote leaf senescence, regulate secondary plant growth, and participate in abiotic stress responses and photomorphogenesis (Tang & Chu, 2020). Genes that have been identified as components of the strigolactone biosynthetic pathway include the cytochrome P450 monooxygenase-encoding *MORE AXILLARY GROWTH 1* (*MAX1*) (Zhang et al., 2018), *CAROTENOID CLEAVAGE DIOXYGENASE 7* (*CCD7*/*MAX3*), and *CAROTENOID CLEAVAGE DIOXYGENASE 8* (*CCD8*/*MAX4*) (Decker et al., 2017). In addition, the gene *MAX2* is involved in strigolactone signal transduction; *MAX2* is an F-box protein that acts as a central regulator in strigolactone signaling.

Strigolactone is mainly synthesized in the root tip (Kohlen et al., 2011). The synthesis process from carotenoid to biologically active SL requires the involvement of at least five enzymes in the following order: β-carotenoid isomerase *DWARF27* (*D27*) (Lin et al., 2009); *carotenoid cleavage dioxygenase 7* (*CCD7*) encoded by the *MAX3/DAD3/RMS5/D17* gene (Zou et al., 2006); *MAX4/ DAD1/RMS1/D10* gene encoding *carotenoid cleavage dioxygenase 8* (*CCD8*) (Sorefan et al., 2003); *MAX1* (*MORE AXILLARY GROWTH 1*) encoding cytochrome P450 monooxygenase (Booker et al., 2005); *LATERAL BRANCHING OXIDOREDUCTASE* (*LBO*), which acts on the CYP711A1 product in non-canonical SLs (Brewer et al., 2016), and CYP722C plays a key role in synthesizing both strigol- and ORO-type canonical SLs for canonical SLs (Wang et al., 2022; Wakabayashi et al., 2020). Mutants of these genes all produce a more-branching phenotype (Lin et al., 2009; Sorefan et al., 2003; Stirnberg, van de Sande & Leyser, 2002; Snowden et al., 2005; Lazar & Goodman, 2005; Arite et al., 2007; Drummond et al., 2009; Waters et al., 2012).

*MAX1* acts downstream of *MAX3* and *MAX4*, of which there is only one copy in Arabidopsis, two of the five *MAX1* homologous genes in rice have been confirmed to be involved in the synthesis of strigolactone (Abe et al., 2014; Seto et al., 2014; Zhang et al., 2014). *MAX1* also has one homologue in tomato, *SlMAX1*/*CYP711A21*, the tomato *Slmax1* mutant has a higher number of branches (Zhang et al., 2018). Knockdown of two *MAX1* homologs in rapeseed using CRISPR/Cas9 can significantly reduce plant height and increase yield (Zheng et al., 2020). *MAX2* can promote a variety of hormones that affect the establishment of plant photomorphogenesis, such as its involvement in the regulation of branching by the *BES1* pathway; in addition, *MAX2* has important roles in responses to abiotic stresses, drought and pathogenic microbes (Chevalier et al., 2014; Wang et al., 2013). Research on *MAX2*-mediated plant growth and development pathways still requires further study.

*MAX3*-encoded *CCD7* acts in chloroplasts. Sequence analysis of homologous genes encoding *CCD7* in Arabidopsis, pea, petunia and rice demonstrated that the regulation mechanism of MAX3 in plant branching is conserved in monocotyledons and dicotyledons (Zou et al., 2006), *MAX4*-encoded *CCD8* may not only involve in the biosynthesis of SLs, but also affect plant reproductive development in other pathways (Kohlen et al., 2012).

Double mutant analysis and reciprocal grafting experiments using *max1*, *max2*, *max3* and *max4* mutants in Arabidopsis demonstrated that the *MAX1*–*MAX4* genes act in the same pathway (Booker et al., 2005), as four key genes in the synthesis and signaling of SLs, knockout mutants exhibit increased tiller number and dwarf phenotypes, although phenotypic differences exist between different *MAXs* genes. Until recently, distribution of *MAX* gene family members in solanaceous species (especially tobacco) had not been reported (Challis et al., 2013).

In the present study, we analyzed the physicochemical properties, conserved regions, phylogeny, cis-acting elements, and expression patterns of the *MAX* gene family. This was accomplished by comparing the genomes of different representative solanaceous crop species including tobacco, tomato, potato, petunia, and pepper. *MAX* gene expression was explored before and after topping treatment in combination with hormone or stress treatments. The results serve as a reference for future in-depth study of *MAX* gene functions in tobacco.

## Materials and Methods

### Identification of *MAX* genes

Genomic information, chromosome locations, protein sequences, and coding sequences (CDSs) of *Nicotiana tabacum* TN90, *Nicotiana tabacum BX*, *Nicotiana tabacum* K326, *Nicotiana sylvestris*, *Nicotiana tomentosiformis*, *Nicotiana benthamiana*, *Solanum lycopersicoides*, *Solanum tuberosum*, *Petunia hybrida*, *Capsicum annuum*, *Arabidopsis thaliana*, and *Oryza sativa* were downloaded from The Sol Genomics Network (https://solgenomics.net). Two methods were used to identify members of the *MAX* gene family. First, the conserved domains of MAX1–MAX4 were downloaded from the Pfam database (http://pfam.xfam.org/; MAX1: PF00067, MAX2: PF18511, MAX3 and MAX4: PF03055) (Czarnecki et al., 2014). These were used as template sequences to identify candidate family members using HMM3.0 (http://hmmer.org/download.html) with an E-value cutoff of 1.0. S, the amino acid sequences of the Arabidopsis MAX1–MAX4 members (AT2G26170.1, AT2G42620.1, AT2G44990.1, AT4G32810.1) were compared within the whole genome using the BLASTP method (threshold E≤1e−10) to obtain candidate members. Candidate family members were manually filtered using SMART (http://smart.embl-heidelberg.de) (PFAM domains) and NCBI (https://www.ncbi.nlm.nih.gov) to obtain the final set of *MAX* gene family members.

### Evolutionary analysis and classification of *MAX* genes

The protein sequences of the identified *MAX* gene family members (Table S1) were compared with known Arabidopsis and rice MAX protein sequences using ClustalW (Larkin et al., 2007). Multiple sequence alignments were displayed with Jalview (Waterhouse et al., 2009). An unrooted phylogenetic tree was constructed using MEGA7.0 (Sudhir, Glen & Koichiro, 2016) with the maximum likelihood method based on the poisson correction model and 1,000 bootstrap replicates, the gap opening penalty was 10.

### Sequence and structural characterization of *MAX* genes

Physicochemical properties such as amino acid number, molecular weight (MW), and theoretical isoelectric point (pI) of MAXs were calculated with ExPASy (https://www.expasy.org/vg/index/Protein). Subcellular localizations were predicted with CELLO v.2.5 (https://mybiosoftware.com/cello-v-2-5-subcellular-localization-predictor.html). Protein sequences of the MAX family were analyzed using MEME (https://meme-suite.org/meme/), and the Gene Structure Display Server (GSDS, v2.0, http://gsds.gao-lab.org/) was used to map *MAX* gene structure.

### Analysis of cis-acting elements of *MAX* promoters

For each tobacco *MAX* gene, the 3-kb region upstream of the transcription start site was extracted with TBtools (Chen et al., 2020) and analyzed with the PLACE database (https://www.dna.affrc.go.jp/PLACE/?action=newplace) and the PlantPAN3.0 database (http://plantpan.itps.ncku.edu.tw/promoter.php). This was done to identify potential transcription factor binding sites and cis-acting elements associated with growth, development, hormone responses, and stress responses.

### Re-analyzing RNAseq dataset of *MAX*

The transcriptome data of root, leaf, flower and fruit (six developmental stages) of tomato MAX genes (The Tomato Genome Consortium, 2012), and the transcriptome data of roots (0, 0.5, 1, 3, 5, 8 and 24 h after topping) of tobacco Yunyan 87 (Qin et al., 2020) MAX genes were analyzed separately. The heatmap was constructed by TBtools.

### Total RNA extraction and fluorescence quantitative PCR (qPCR)

*Nicotiana tabacum* L. cv. ‘honghuadajinyuan’ was grown in the greenhouse. The seeds were obtained from the Tobacco Research Institute (TRI) of the Chinese Academy of Agricultural Sciences (CAAS). Total RNA was extracted from the roots, stems, leaves, flowers, apical buds, and axillary buds during the vigorous growth period using the GeneJET™ Plant RNA Purification Mini Kit (MBI Fermentas, Burlington, Canada). Samples were run on 1% agarose gels and purity was assessed with a NanoDrop2000 spectrophotometer. Total RNA was reverse transcribed using the RevertAid™ First-Strand cDNA Synthesis Kit (MBI Fermentas, Burlington, Canada). Quantitative reverse transcription (qRT)-PCR and qPCR were performed with the resulting cDNA and TB Green™ Premix Ex Taq™ II (TliRNaseH Plus) (TaKaRa, Japan) with primers specific to the candidate genes (Table 1). Actin gene was selected as the internal reference gene for normalization. Each sample was analyzed with three replicates. The relative expression levels were calculated using the 2^ $-\rm \Delta \Delta Ct$^ method (Livak & Schmittgen, 2002).

**Table 1:** Primer sequences for genes used in qRT‑PCR.

Name	Sequence (5′–3′)	
	F	R
NtMAX1	TGGCTCTTGGAGTTCTTGCT	TCCATAAGGGGAGAGTGTCG
NtMAX2	AGGTGAGGCATCAAGCAACT	AAGCTCACCAACACCAATCC
NtMAX3	TCCTGAAAGGTGGGAAGATG	GGGCTTCAAGATTTGAGCAG
NtMAX4	ACCATTCTTTGTGCCTCGAC	TGCAGTCCATAGGGAAGACC
NtActin	CAAGGAAATCACCGCTTTGG	AAGGGATGCGAGGATGGA

### Hormone, topping, and stress treatments

Hormone treatments were applied to *N. tabacum* L. cv. ‘honghuadajinyuan’ during the vigorous growth period. These treatments comprised 0.1 mmol/l abscisic acid (ABA) (Wang & Qiang, 2012), 0.0001 mol/l indole-3-acetic acid (IAA) (Vehn & Sauer, 2017), 300 mM sucrose, 50 µM of the synthetic strigolactone analog GR24 (Fan et al., 2021), and 200 µM 6-benzylaminopurine (6-BA) (Wang et al., 2017) according to published references. The axillary buds of the first leaf were also sampled before topping and 1, 3, and 5 d after topping according to published references (Wang et al., 2018). Plants were treated with simulated drought stress (260 µmol L-1 mannitol) or low temperature stress (4 °C) then sampled at 1, 2, 4, and 8 d (Zhang et al., 2015) or at 1 and 2 d (Gu et al., 2021), respectively. There were three biological replicates for each treatment. The results were analyzed with TBtools and Sigmaplot 10.0 software.

### Subcellular localization analysis of representative MAX proteins in tobacco

CDSs of NtMAX2, NtMAX3, and NtMAX4 were each cloned with the stop codon removed, then inserted into the PYG57 vector using the restriction enzymes SacI and SpeI. The recombinant vectors and empty plasmid were each transformed into *Agrobacterium tumefaciens* EHA105. Four-day-old leaves of *Nicotiana benthamiana* were inoculated with transformed *Agrobacterium* EHA105 *via* needle prick. Plants were then incubated continuously at 28 °C with a 16/8 h light/dark cycle. Leaves were photographed using a Leica TCS SP8 laser confocal microscope (Leica, Mannheim, Germany).

## Results

### Identification of *MAX* genes

A total of 74 non-redundant MAX family proteins were identified in tobacco, tomato, potato, petunia, and pepper. The gene ID, protein length, molecular weight, isoelectric point, and subcellular localization of 89 MAX family members (including in Arabidopsis and rice) are shown in Table 2. PI values of MAX1 homologs were all in the alkaline range, whereas PI values of the MAX2 and MAX4 homologs were all in the acidic range. It is noteworthy that for the selected solanaceous crops, members of the MAX1, MAX2, and MAX3/MAX4 sub-families were mainly localized in the mitochondria, nucleus, and cytoplasm, respectively. This implies that different sub-types of MAX proteins may perform different functions. Table 3 shows the number of MAX sub-family members predicted in different species, as well as the ranges of PI and MW values and protein lengths. The results showed that the MAX family was not large in either monocotyledons or dicotyledons; *Nicotiana sylvestris* contained the largest MAX4 sub-family, with 16 members, but whether these 16 homologous genes have functional redundancy needs further experimental verification.The distribution of genes within each sub-family was similar between species.

**Table 2:** *MAX* gene family members in representative solanaceous crops.

		Protein				MAX1
Species	Gene ID	Length	MW (k Da)	PI	Subcellular localization	
*Nicotiana tabacum* (K326)	mRNA_49921	467	53.08	8.79	Mitochondrial	
*Nicotiana tabacum* (TN90)	mRNA_42371	559	63.55	9.02	PlasmaMembrane/Mitochondrial	
*Nicotiana tabacum* (BX)	mRNA_49611	559	63.55	0.02	PlasmaMembrane/Mitochondrial	
*Nicotiana tomentosiformis*	/	/	/	/		
*Nicotiana sylvestris*	mRNA_44016	560	63.52	8.95	PlasmaMembrane/Mitochondrial	
*Nicotiana benthamiana*	Niben101Scf01777g03001.1	515	58.52	8.91	Mitochondrial	
*Solanum lycopersicum*	Solyc08g062950.2.1	519	58.71	8.85	Mitochondrial	
*Arabidopsis thaliana*	AT2G26170.1	522	59.43	9.31	PlasmaMembrane/Mitochondrial	
*Oryza sativa*	LOC_Os01g50530.1	411	46.79	8.56	Mitochondrial/Cytoplasmic	
	LOC_Os01g50580.1	385	43.2	5.66	Cytoplasmic	
	LOC_Os01g50590.1	516	57.96	7.75	Mitochondrial	
	LOC_Os02g12890.1	548	60.56	8.55	Mitochondrial/PlasmaMembrane	
	LOC_Os02g12890.2	423	46.73	7.17	Cytoplasmic/Mitochondrial	
	LOC_Os06g36920.1	549	60	9.63	Mitochondrial	
*Solanum tuberosum*	PGSC0003DMT400014456	519	58.66	8.75	Mitochondrial	
*Petunia hybrida*	Peaxi162Scf00327g00049.1	533	60.47	9.1	Mitochondrial	
*Capsicum annuum*	CA08g05010	534	60.11	9.23	PlasmaMembrane/Mitochondrial	
*Nicotiana tabacum* (K326)	mRNA_29422	726	81.06	5.74	Nuclear/Cytoplasmic	MAX2
	mRNA_29423	726	81.06	5.74	Nuclear/Cytoplasmic	
	mRNA_29424	726	81.06	5.74	Nuclear/Cytoplasmic	
*Nicotiana tabacum* (TN90)	mRNA_20815	726	81.06	5.74	Nuclear/Cytoplasmic	
	mRNA_20816	726	81.06	5.74	Nuclear/Cytoplasmic	
	mRNA_20817	726	81.06	5.74	Nuclear/Cytoplasmic	
	mRNA_20818	726	81.06	5.74	Nuclear/Cytoplasmic	
*Nicotiana tabacum* (BX)	/	/	/	/		
*Nicotiana tomentosiformis*	mRNA_48826	352	39.51	5.52	Nuclear/Extracellular	
*Nicotiana sylvestris*	mRNA_67458_cds	727	81.03	5.74	Nuclear/Cytoplasmic	
	mRNA_67459_cds	727	81.03	5.74	Nuclear/Cytoplasmic	
	mRNA_67460_cds	727	81.03	5.74	Nuclear/Cytoplasmic	
*Nicotiana benthamiana*	Niben101Scf00536g02005.1	601	66.77	5.66	Cytoplasmic/Nuclear	
	Niben101Scf01068g01004.1	683	75.85	5.94	Nuclear/Extracellular	
	Niben101Scf04933g00008.1	683	75.74	5.53	Extracellular	
	Niben101Scf09559g00005.1	726	80.89	5.7	Cytoplasmic/Extracellular/Nuclear	
	Niben101Scf10248g01017.1	561	62.33	5.85	Cytoplasmic/Nuclear	
*Solanum lycopersicum*	Solyc07g055120.2.1	676	75.74	5.55	Nuclear/Extracellular	
	Solyc12g010900.1.1	722	80.74	5.94	Extracellular	
*Arabidopsis thaliana*	AT2G42620.1	693	77.42	5.36	Nuclear/PlasmaMembrane	
*Oryza sativa*	LOC_Os06g06050.1	720	79.23	5.33	Cytoplasmic/PlasmaMembrane	
*Solanum tuberosum*	PGSC0003DMT400013844	723	80.71	5.73	PlasmaMembrane/Cytoplasmic	
	PGSC0003DMT400057424	711	79.42	5.4	Nuclear/Extracellular	
*Petunia hybrida*	Peaxi162Scf00469g00034.1	561	62.65	6.1	Cytoplasmic/Nuclear/Extracellular	
	Peaxi162Scf00384g00211.1	695	77.77	5.85	Extracellular/Cytoplasmic	
*Capsicum annuum*	CA09g13650	721	80.84	6.1	Cytoplasmic/Nuclear	
	CA00g44080	706	78.9	5.05	PlasmaMembrane/Nuclear	
*Nicotiana tabacum* (K326)	mRNA_108065	157	17.66	8.55	Cytoplasmic/Chloroplast	MAX3
	mRNA_108066	336	37.91	6.08	Extracellular	
	mRNA_108067	651	73.27	6.43	Cytoplasmic	
	mRNA_34367	256	28.88	5.59	Cytoplasmic	
*Nicotiana tabacum* (TN90)	mRNA_51017	651	73.27	6.43	Cytoplasmic	
	mRNA_51018	336	37.91	6.08	Extracellular	
*Nicotiana tabacum* (BX)	mRNA_110488	295	32.97	8.75	Mitochondrial/Cytoplasmic	
*Nicotiana tomentosiformis*	mRNA_68553	651	73.19	6.43	Cytoplasmic	
*Nicotiana sylvestris*	\	\	\	\		
*Nicotiana benthamiana*	Niben101Scf00878g02006.1	651	73.8	6.43	Cytoplasmic	
*Solanum lycopersicum*	Solyc01g090660.2.1	663	75.03	7.25	Cytoplasmic	
*Arabidopsis thaliana*	AT2G44990.1	629	70.86	6.3	Nuclear/Cytoplasmic	
*Oryza sativa*	LOC_Os04g46470.1	609	68.19	9.19	Mitochondrial	
*Solanum tuberosum*	PGSC0003DMT400045162	645	72.88	6.61	Cytoplasmic	
*Petunia hybrida*	Peaxi162Scf00377g00829	621	69.94	5.96	Cytoplasmic	
*Capsicum annuum*	CA00g32540	646	72.73	6.27	Cytoplasmic	
*Nicotiana tabacum* (K326)	mRNA_112609	555	62.2	6.18	Cytoplasmic	MAX4
	mRNA_141629	556	62.35	5.98	Cytoplasmic	
*Nicotiana tabacum* (TN90)	mRNA_89885	556	62.35	5.98	Cytoplasmic	
	mRNA_98197	555	62.2	6.18	Cytoplasmic	
*Nicotiana tabacum* (BX)	mRNA_104099	556	62.35	5.98	Cytoplasmic	
	mRNA_112966	555	62.2	6.18	Cytoplasmic	
*Nicotiana tomentosiformis*	mRNA_52451	556	62.19	6.3	Cytoplasmic	
*Nicotiana sylvestris*	mRNA_11465_cds	357	40.35	5.31	Cytoplasmic	
	mRNA_11466_cds	375	42.34	5.53	Cytoplasmic	
	mRNA_11467_cds	368	41.65	5.53	Cytoplasmic	
	mRNA_11468_cds	557	62.5	6.34	Cytoplasmic	
	mRNA_11469_cds	444	50.18	6.21	Cytoplasmic	
	mRNA_11470_cds	442	49.96	6.21	Cytoplasmic	
	mRNA_51723_cds	601	68.13	5.99	Cytoplasmic	
	mRNA_51724_cds	585	66.44	5.96	Cytoplasmic	
	mRNA_8034_cds	441	49.6	6.11	Cytoplasmic	
	mRNA_8035_cds	547	61.15	6.02	Cytoplasmic	
	mRNA_8036_cds	441	49.6	6.11	Cytoplasmic	
	mRNA_8037_cds	562	62.93	5.93	Cytoplasmic	
	mRNA_8038_cds	441	49.6	6.11	Cytoplasmic	
	mRNA_8039_cds	441	49.6	6.11	Cytoplasmic	
	mRNA_8040_cds	441	49.6	6.11	Cytoplasmic	
	mRNA_85696_cds	601	65.7	6.62	Chloroplast	
*Nicotiana benthamiana*	Niben101Scf01056g05003.1	127	14.7	5	PlasmaMembrane/Chloroplast	
	Niben101Scf01611g07010.1	555	62.36	5.98	Cytoplasmic	
*Solanum lycopersicum*	Solyc08g066650.2.1	557	62.08	6.37	Cytoplasmic	
*Arabidopsis thaliana*	AT4G32810.1	570	63.96	6.65	Cytoplasmic	
*Oryza sativa*	LOC_Os01g38580.1	552	59.92	5.88	Chloroplast	
	LOC_Os01g38580.2	506	54.88	6.29	Chloroplast	
	LOC_Os01g54270.1	801	87.95	5.89	Chloroplast/Mitochondrial	
*Solanum tuberosum*	\	\	\			
*Petunia hybrida*	Peaxi162Scf00227g00714.1	556	62.1	6.33	Cytoplasmic	
*Capsicum annuum*	CA11g14280	534	56.15	6.04	Cytoplasmic	

**Table 3:** The number of *MAX1*–*MAX4* subfamily members in selected species.

	K326	TN90	BX	Ntom	Nsyl	Niben	Tomato	Potato	Petunia	Pepper	Arabidopsis	Rice	Length	MW (k Da)	PI
MAX1	1	1	1	/	1	1	1	1	1	1	1	6	467–560	53.08–63.55	8.79–9.23
MAX2	3	4	/	1	3	5	2	2	2	2	1	1	352–727	39.51–81.06	5.05–6.1
MAX3	4	2	1	1	/	1	1	1	1	1	1	1	157–663	17.66–75.03	5.59–8.75
MAX4	2	2	2	1	16	2	1	/	1	1	1	3	127–601	14.7–68.13	5–6.62

### Phylogenetic and structural analysis of MAX genes

**Figure 1:**
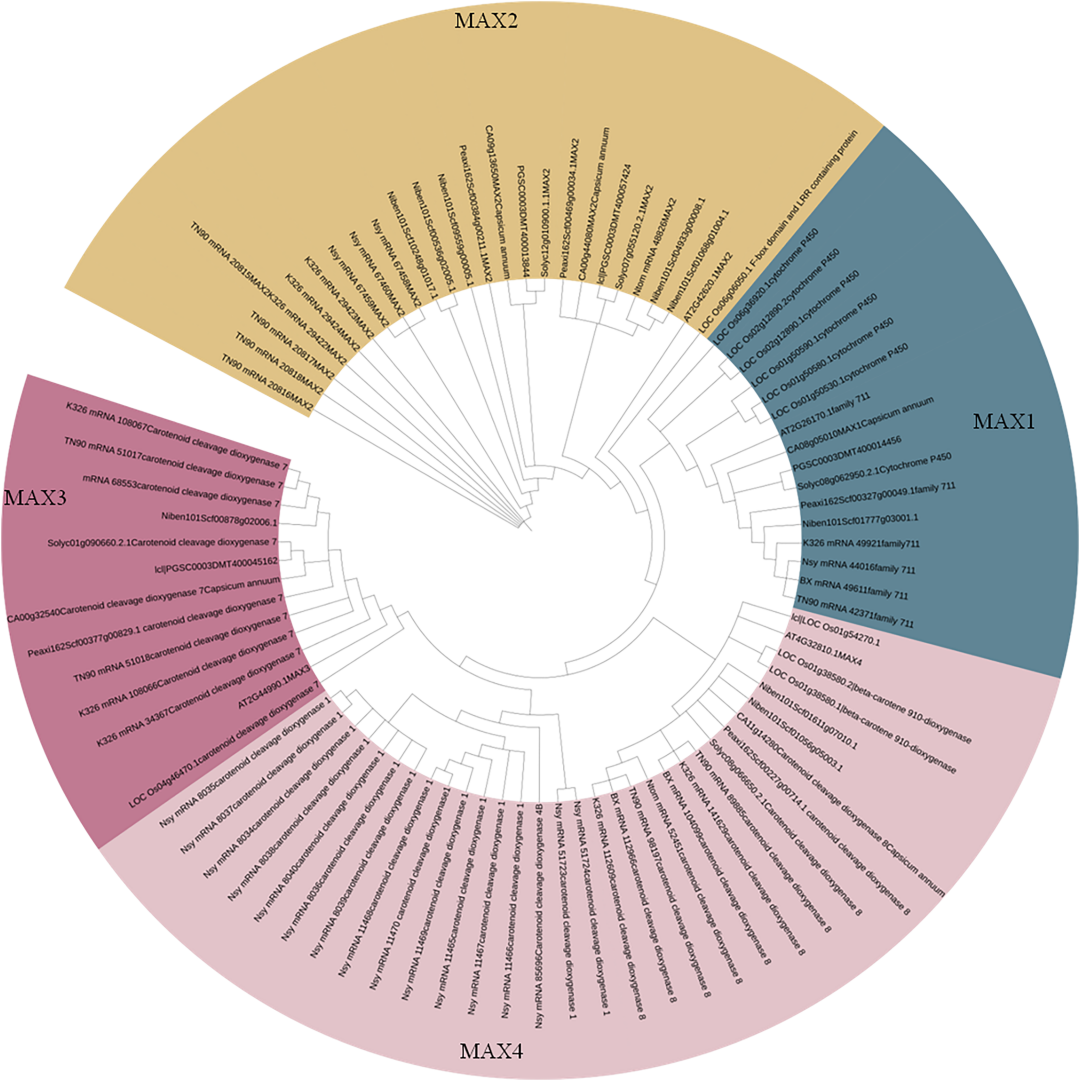
Phylogenetic analysis of MAX family members.

**Figure 2:**
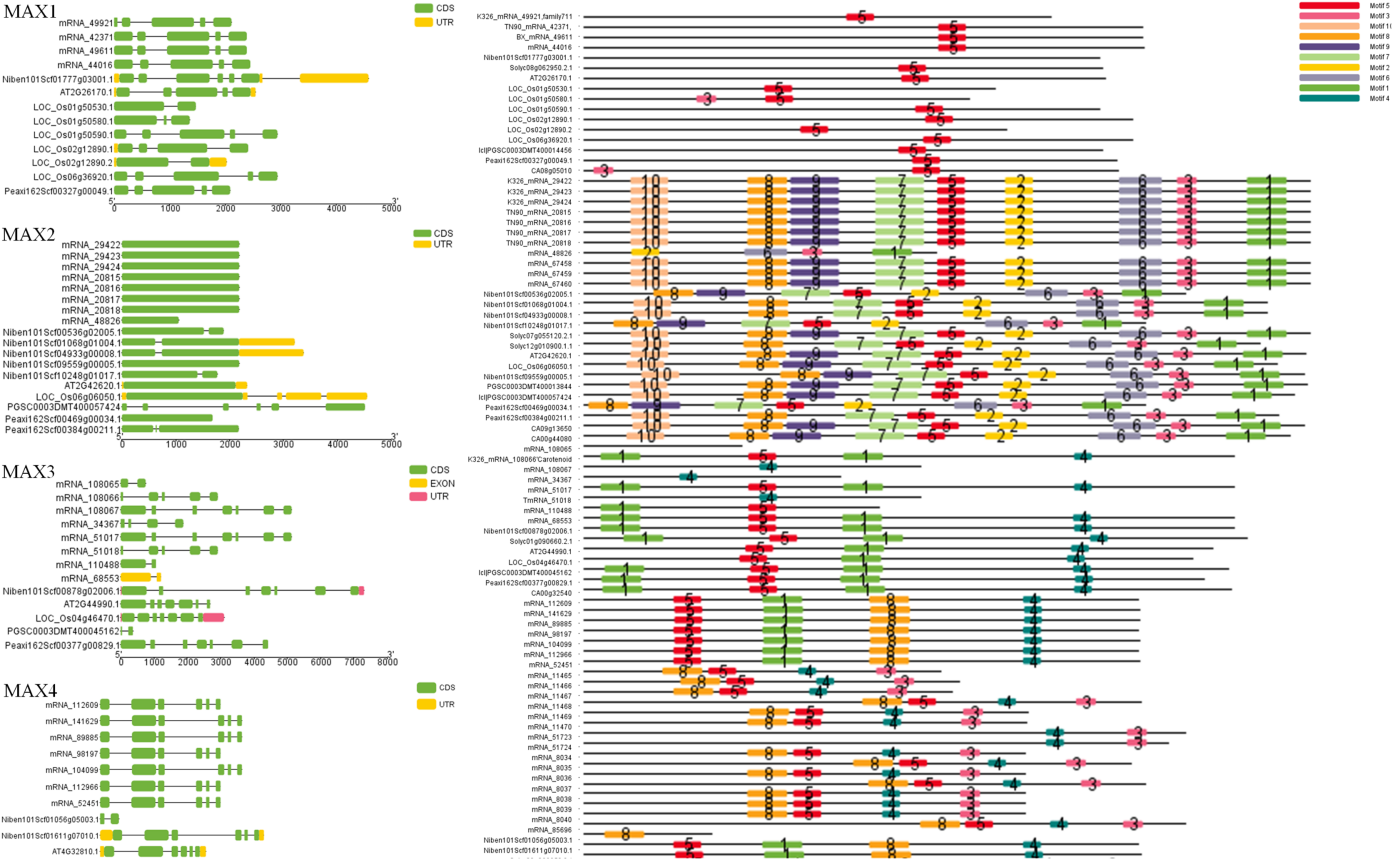
MAX gene exon–intron structures and sequence motifs.

**Figure 3:**
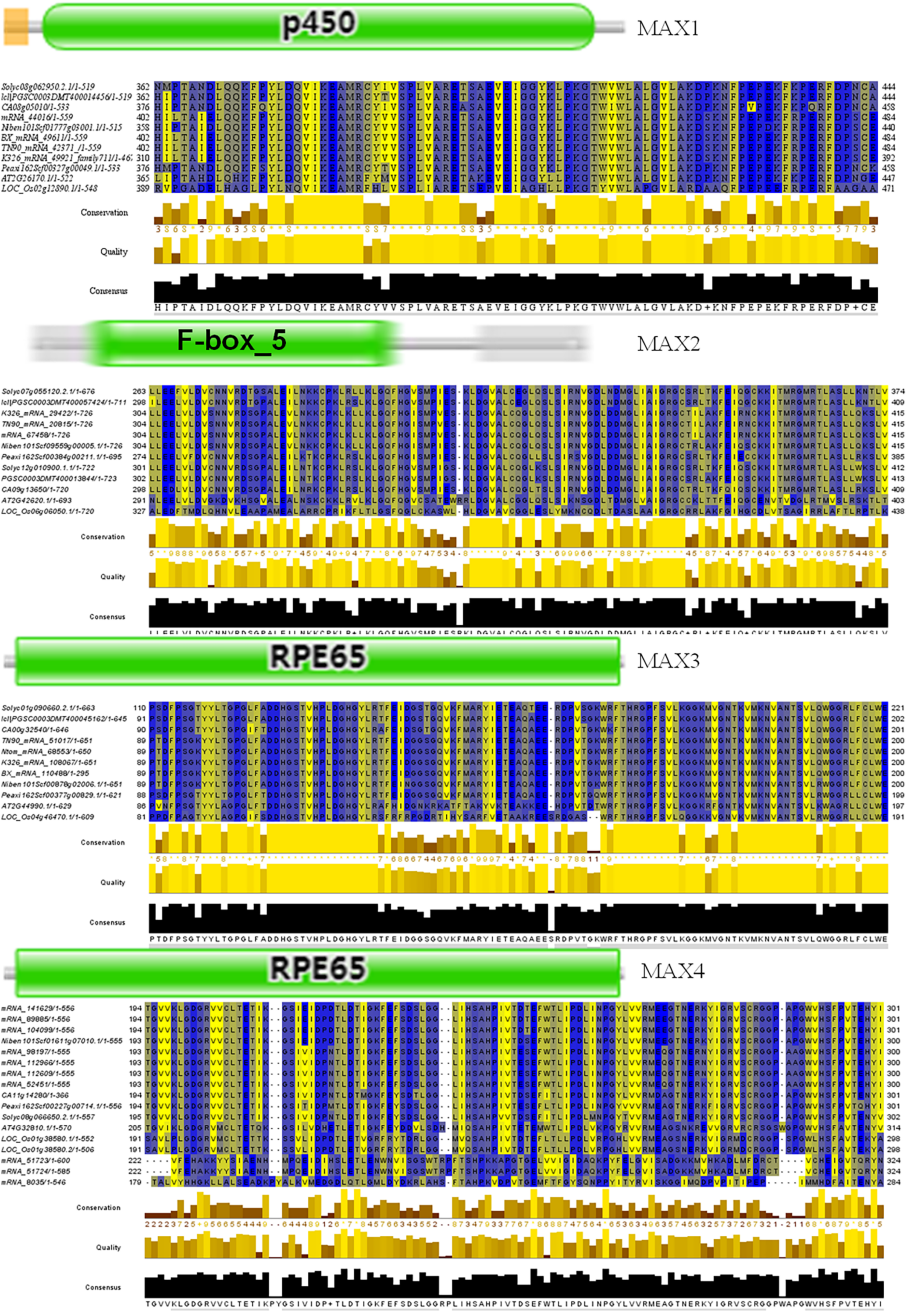
Conserved motifs of representative MAX proteins in solanaceous crops.

To better understand the evolutionary relationships between MAX family members, an unrooted phylogenetic tree was constructed using the full-length sequences of 87 MAX1–MAX4 proteins (Fig. 1); two sequences (mRNA_108065 and mRNA_110488) were removed during construction of the evolutionary tree due to their relatively long distances from other sequences. Based on maximum likelihood phylogenetic analysis, the MAX1–MAX4 sub-families contained 16, 26, 13, and 32 members, respectively. The sequences of the MAX family members are listed in Table S1. The exon–intron structures and conserved motifs of the MAX genes were analyzed (Fig. 2). There were at least two exons in all members of the MAX1, MAX3, and MAX4 sub-families, with maximum exon numbers of five, seven, and nine, respectively. In contrast, members of the MAX2 family contained only one to two exons each, except for PGSC0003DMT400057424, which contained six. MEME analysis of MAX protein sequences revealed the existence of sub-family-specific motifs. For example, motifs 2, 6, 7, 9, and 10 were uniquely found in MAX2 proteins, and motif 4 was specific to MAX3 and MAX4 genes. In contrast, motif 5 was common to the MAX1–MAX4 sub-families. The results showed that each sub-family had a unique and consistent gene structure with conserved motifs. Alignment of representative MAX1–MAX4 proteins showed that they all contained typical conserved domains (Fig. 3 and Fig. S1).

### Analysis of cis-acting elements and transcription factor binding sites in the promoter region of the tobacco MAX family

To investigate the potential biological functions of MAX genes in tobacco, the 3-kb promoter sequences of MAX1–MAX4 gene family members were analyzed using the PlantCARE database. Cis-acting elements associated with meristem development and responses to hormones, sucrose, light, and stress were screened (Table S2). We found that ASF1MOTIFCAMV, CPBCSPOR, ERELEE4, GAREAT, MYBGAHV, and TATCCAOSAMY (which are closely associated with responses to auxin, cytokinin, ethylene, gibberellin (GA), and sucrose) are widely distributed in the tobacco MAX promoters, consistent with previous observations. It has been demonstrated that strigolactone can inhibit plant branching by inducing cytokinin and auxin to promote axillary bud dormancy, and can inhibit stem elongation by acting on photoreceptors and auxin (Yao, Li & Xie, 2018; Luo et al., 2019; Jia et al., 2014; Xiong, Wang & Li, 2014). Recent discoveries indicate that SL biosynthesis is regulated by GAs (Marek, 2017) and that SLs can induce ethylene to elongate root hairs (Kapulnik et al., 2011). Sucrose can promote shoot branching by suppressing the inhibitory effect of SL (Patil et al., 2021).

In recent years, it has been found that SLs play key roles in response to abiotic stresses such as drought, high salt, and nutrient deficiencies in plants (Visentin et al., 2016). In tomato, strigolactone biosynthesis is induced by heat and cold stresses (Chi et al., 2021). This is consistent with our finding that a large number of cis-elements associated with cold and other stress responses exist in *MAX* gene promoters. Many cis-acting elements related to pathogenesis and wounding induction were also identified, suggesting that SL may have functions in disease resistance, which is in line with previous research (Torres-Vera et al., 2014). These results imply that the cis-elements in the promoter sequences of *MAX1*/*3*/*4*, which are involved in SL biosynthesis, and *MAX2*, which is involved in SL signal sensing and transduction, are together able to ensure appropriate SL functioning with respect to plant branching and responding to cues such as light and hormones.

Previous transcriptome analysis revealed that genes responsive to SL include TCP, NAC, WRKY, and MYB transcription factors (Tang & Chu, 2020; Wang et al., 2021). We analyzed and counted potential transcription factor binding sites in *MAX* family members of three tobacco cultivars (*N. tabacum* K326, KN90, and BX), two ancestral species of cultivated tobacco (*Nicotiana sylvestris* and *Nicotiana tomentosiformis*), and triploid *Nicotiana benthamiana* (Table 4). We found that the number of transcription factor binding sites varied widely between tobacco species with different ploidy levels. This was especially true of AP2, MYB, TCP, and WRKY transcription factors in *MAX1* homologs; AP2, bHLH, bZIP, and TCP in *MAX2* homologs; AP2, MYB, and TCP in *MAX3* homologs; and AP2, bHLH, bZIP, and MYB in *MAX4* homologs. Prior studies have noted the importance of AP2 transcription factors in responding to drought, low temperature, and high salt in plants (Xie et al., 2019). Based on these data, we can infer that *MAX* family members play important roles in abiotic stress responses.

**Table 4:** Number of potential transcription factor binding sites in the 3,000-bp promoter region of *MAX* genes in tobacco.

	K326	TN90	BX	Ntom	Nsy	Niben	
AP2	194	100	219	/	219	209	MAX1
B3	72	83	75	/	74	60	
bHLH	35	67	133	/	134	52	
bZIP	88	97	121	/	123	72	
C2H2	17	15	21	/	24	13	
MYB	143	168	157	/	151	152	
NAC; NAM	38	14	11	/	12	16	
TCP	174	216	148	/	152	99	
WRKY	123	110	37	/	37	41	
Aux/IAA	0	0	1	/	1	0	
AP2	130	130	/	193	162	161	MAX2
B3	56	69	/	71	75	60	
bHLH	120	117	/	89	145	77	
bZIP	127	128	/	150	146	107	
C2H2	20	15	/	18	21	25	
MYB	72	62	/	143	75	129	
NAC; NAM	10	9	/	25	10	9	
TCP	184	126	/	188	148	121	
WRKY	32	18	/	59	19	51	
Aux/IAA	1	1	/	1	1	1	
AP2	220	208	212	172	/	63	MAX3
B3	56	51	48	47	/	9	
bHLH	68	91	131	74	/	12	
bZIP	104	142	145	129	/	21	
C2H2	26	29	23	19	/	1	
MYB	144	136	130	168	/	18	
NAC; NAM	16	11	18	14	/	8	
TCP	112	153	136	179	/	37	
WRKY	54	65	84	122	/	10	
Aux/IAA	1	2	1	2	/	0	
AP2	125	206	205	195	169	119	MAX4
B3	38	76	77	82	74	39	
bHLH	180	234	233	264	128	120	
bZIP	127	177	177	175	137	117	
C2H2	15	15	15	11	16	9	
MYB	142	154	154	148	155	101	
NAC; NAM	23	25	25	27	14	12	
TCP	62	168	167	239	133	50	
WRKY	52	65	65	115	46	44	
Aux/IAA	1	2	2	1	1	1	

### Tissue-specific *MAX* expression analysis in tobacco

**Figure 4:**
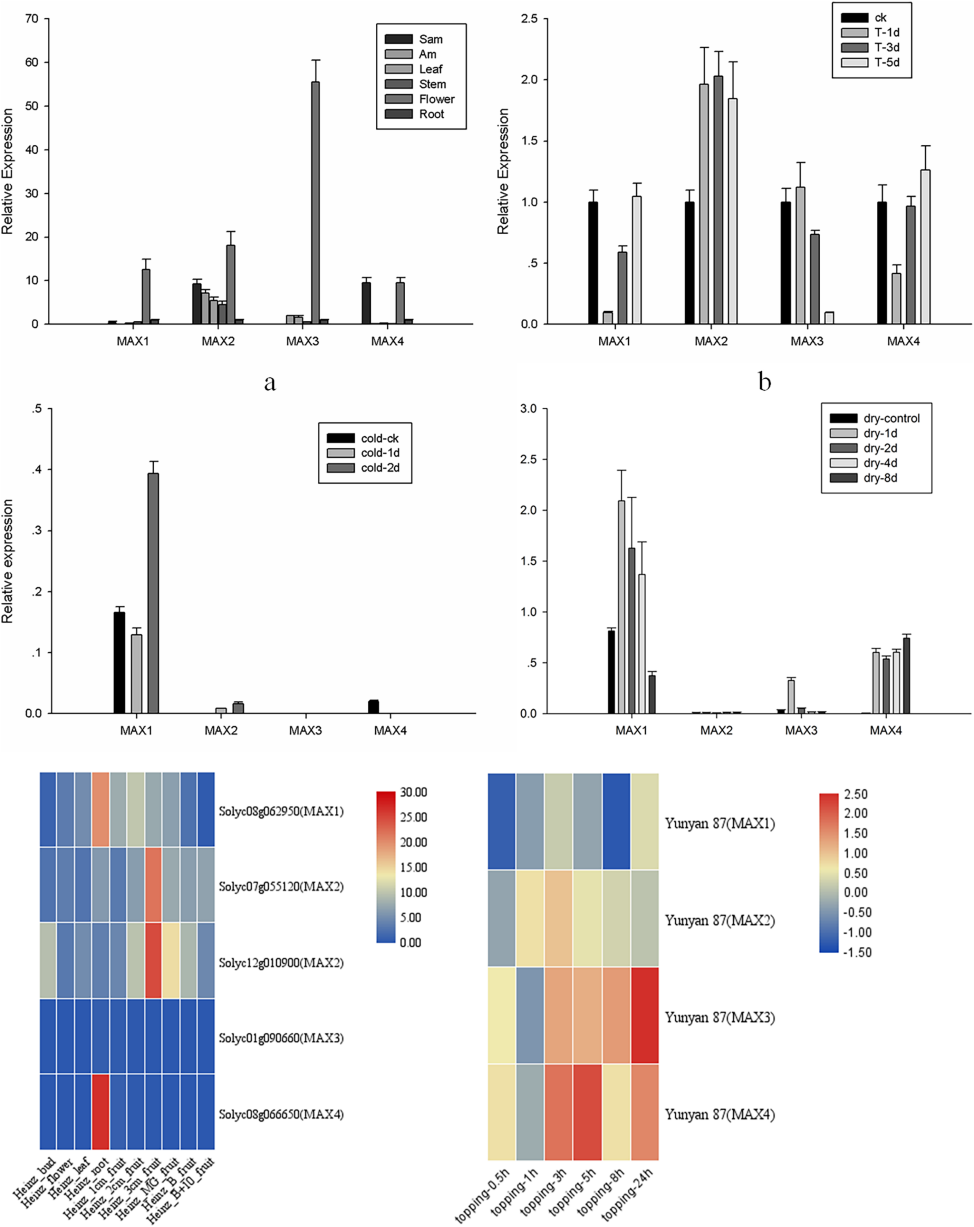
*MAX1*–*MAX4* gene expression in tobacco. (A) *MAX* gene expression in different tissues. (B) *MAX* gene expression in response to topping treatment. (C) *MAX* gene expression in response to cold treatment. (D) *MAX* gene expression in response to drought. (E) Heatmap of tomato *MAX* Members in different tissues. (F) Heatmap of *MAX* members within 24 h after topping in Yunyan 87.

To analyze the roles of *MAX* genes in the growth and development of tobacco, tissue-specific expression analysis was performed for *MAX1*–*MAX4* genes in apical buds, axillary buds, leaves, stems, flowers, and roots of cultivated tobacco (Fig. 4A). All *MAX* genes were highly expressed in the flowers, with *MAX1* and *MAX3* specifically expressed in flowers and *MAX*4 specifically expressed in apical buds and flowers. Expression trends were similar for *MAX1*/*3*/*4* sub-family members, whereas *MAX2* members were expressed in all tissues, which is consistent with Arabidopsis (Stirnberg, Furner & Leyser, 2007). The tomato transcriptome data indicated that *MAX1* and *MAX4* have the highest expression in tomato roots, while *MAX2* has the highest expression in fruits, which indicates that tomato *MAX2* may play vital roles in fruit ripening, however *MAX3* has very low expression in different tissues (Fig. 4E). All the raw data for fluorescent quantitative PCR is listed in Table S3.

### *MAX* gene expression analysis in response to topping, stress, and hormone treatments

An important physiological role of strigolactone is to regulate branching by interacting with other plant hormones. Tobacco production in the field requires topping treatments, which lead to axillary buds due to the loss of apical dominance. To further analyze the role of *MAX* genes in the responses of tobacco to topping, stress, and hormone treatments, expression levels of *MAX1*–*MAX4* were analyzed at 1, 3, and 5 d after topping or hormone application, at 1, 2, 4, and 8 d after drought treatment, and at 1 and 2 d after cold treatment. The expression level of *MAX2* and *MAX3* after topping rises and then falls, in contrast to the expression level of *MAX1* and *MAX4* after topping, which falls and then rises (Fig. 4B). These results differ from the expression pattern of Yunyan 87, in which within 24 h after topping, *MAX1* and *MAX2* rise first and then fall, while the expression levels of *MAX3* and *MAX4* falls and then rise (Fig. 4E), indicating that *MAX* genes differentially respond to topping in different tobacco varieties.

*MAX* family members were more sensitive to drought than to cold treatment in cultivated tobacco, with a significant increase in expression of *MAX1*/*3*/*4* after drought treatment (Figs. 4C and 4D). Despite the low expression, *MAX2* expression level changed after drought treatment, a finding that is similar to the results of previous studies, suggesting that the *max2* mutant in *Arabidopsis* was sensitive to drought stress (Bu et al., 2014). However, the sensitivity of *max2* was significantly lower under drought treatment compared to *max3* and *max4* mutant plants (Chien et al., 2014), and the sensitivity of *MAX2* to drought was further reduced in tobacco compared to *Arabidopsis*. Unraveling the molecular evolutionary mechanisms behind this interesting phenomenon will be a future focus of our research. The data suggest that SLs may have varying regulatory mechanisms in response to drought stress in different species, nonetheless, a number of questions remain to be answered regarding the diversity and function of SLs.

**Figure 5:**
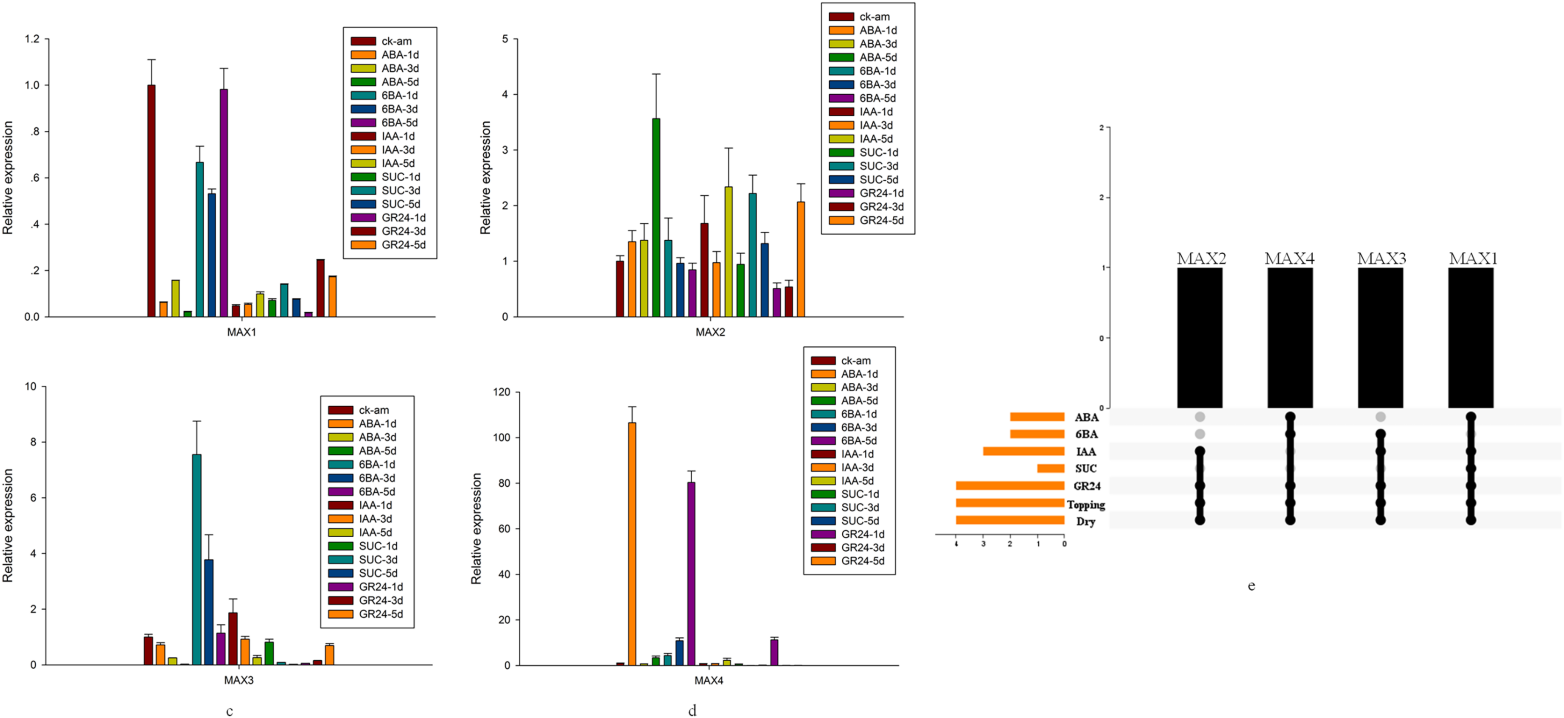
*MAX* gene expression patterns in response to several treatments. (A) Expression levels of *MAX1*in response to hormone treatment. (B) Expression levels of *MAX2*in response to hormone treatment. (C) Expression levels of *MAX3* in response to hormone treatment. (D) Expression levels of *MAX4* in response to hormone treatment. (E) Summary of hormone/stress-induced *MAX* genes. Black dots indicate *MAX* genes that are responsive to hormone/stress treatments, and gray dots indicate *MAX* genes that did not respond to the treatments.

Detailed analysis was conducted to measure the expression levels of *MAX1*–*MAX4* genes in response to ABA, 6-BA, IAA, sucrose, GR24, and topping treatments (Figs. 5A–5D). From these data, we reached several general conclusions. First, *MAX1*–*MAX4* genes in cultivated tobacco all responded positively to GR24 and topping treatments. Second, *MAX1*, *MAX3*, and *MAX4*, which are involved in SL biosynthesis, did not have consistent responses to each treatment; only *MAX4* responded to sucrose, whereas both *MAX3* and *MAX4* responded to IAA, and *MAX2*–*MAX4* responded to both 6-BA and drought treatment (Fig. 5E).

### Subcellular localization of MAX proteins in tobacco

**Figure 6:**
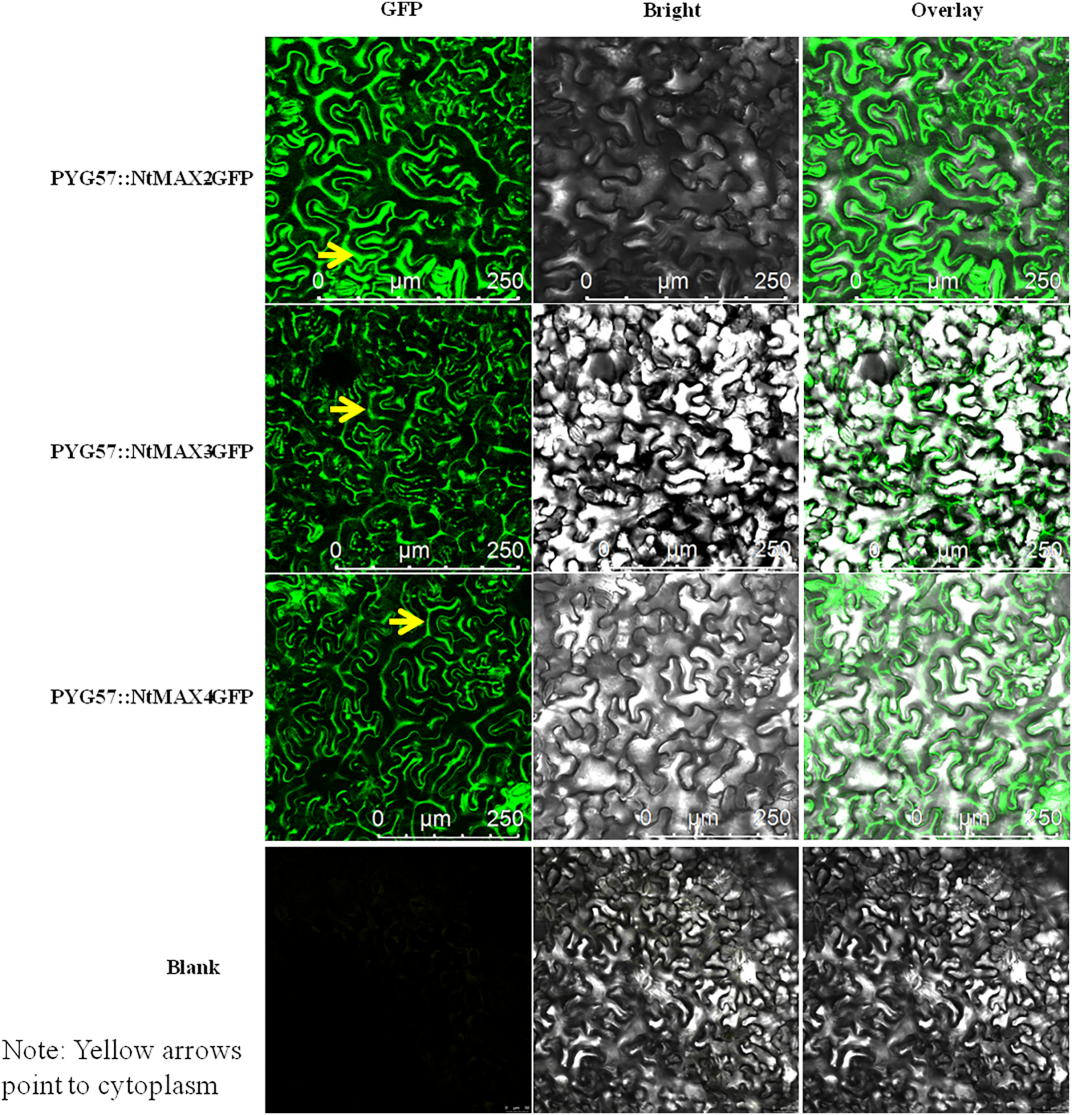
Subcellular localization of NtMAX2/3/4 in tobacco.

The fusion vectors PYG57::NtMAX2-GFP, PYG57::NtMAX3-GFP, and PYG57::NtMAX4-GFP were constructed and co-cultured in tobacco leaves using an *Agrobacterium tumefaciens* EHA105-mediated transformation method. The subcellular localization of each target protein was indicated by green fluorescence of the fusion protein (Fig. 6). There was no green fluorescence in the plasma membrane, cytoplasm, or nucleus of leaves injected with the control vector. MAX2 was localized to the cytoplasm, which was inconsistent with the result predicted by CELLO. MAX3 and MAX4 were also localized to the cytoplasm, which was consistent with the predicted results.

## Discussion

In this study, a total of 74 MAX gene family members, including 9 MAX1, 24 MAX2, 13 MAX13 and 28 MAX4, were identified in the whole genome of a representative solanaceous crop by bioinformatics and genomics. The copy numbers and amino acid length of MAX in different species is consistent with previous reports (Cheng et al., 2018; Dong et al., 2013). In terms of the distribution of introns, the number of introns in the MAX gene differs markedly between monocots and dicots, with MAX1 having 4–5 introns in dicots and 1–4 in monocots; MAX2 having 0–5 introns in dicots and 0–3 in monocots; MAX3 having 1–6 introns in dicots and 6 in monocots; and MAX4 having 1–5 introns in dicots and 5–8 introns in monocots. In monocotyledonous rice, intron-rich genes are usually expressed at higher levels (Deshmukh, Sonah & Singh, 2016), so for MAX3 and MAX4, the higher number of introns in monocotyledonous plants may lead to higher MAX3/4 expression levels, However for most eukaryotes, the loss of introns is a common phenomenon in evolution (Lin et al., 2006; Hooks, Delneri & Griffiths, 2014), and whether this variability in intron distribution is related to the different evolutionary pathways of MAX in mono- and dicotyledons requires further validation.

A 3,000 bp sequence from the upstream of the MAX gene start codon was selected from the published tobacco genome sequence and used for cis-acting element prediction. Based on the results, a large proportion of the cis-acting elements were the meristem development response elements, the hormone (sucrose) response elements, the stress response elements and the light response elements (Table S2). Based on the predicted transcription factor binding sites (Table 4), the large number of TCP, WRKY, and AP2 also suggests that tobacco MAX genes play an vital role in the regulation of meristem development and stress resistance, Wang et al. (2020) identified 401 SLs-responsive genes in *A. thaliana* using synthetic SLs, confirming that they regulate plant branching, leaf shape, drought adaptation and anthocyanin accumulation mainly through transcription factors such as *BRC1*, *TCP1* and *PAP1*. Their data is consistent with our predicted results for transcription factors that bind the MAX promoter in tobacco. The fluorescent quantitative PCR results confirmed that various subclasses of tobacco MAX do respond differently to drought, cold treatment, hormone/sucrose treatment and topping treatment. The diversity of plant promoter cis-elements and the variety of transcription factors that bind to promoters make the mechanism of the promoter a complex process, and provides the questions as to: How do the various elements of the tobacco MAX promoter perform their functions? What is the evolutionary role of the specific cis-elements of the promoter? These questions need to be further explored.

SLs implement functions in plants mainly through complex interactions with other hormones, and previous studies have shown that SLs and auxins can co-regulate branching (Shen et al., 2012). In rice, pea and *Arabidopsis*, transcript levels of *MAX3* and *MAX4* are significantly up-regulated by IAA (Hayward et al., 2009), which is consistent with our results in tobacco. SLs influence root development through the cytokinin signaling network pathway or by interacting with ethylene, auxin and gibberellin (Jiang et al., 2016). Similar to tobacco, *MAX1* in rice and Arabidopsis can also respond to abscisic acid (Marzec & Muszynska, 2015), while unlike rose, where *RwMAX1* and *RwMAX2* can both respond to sucrose, but only *MAX1* in tobacco responds to sucrose treatment (Azri et al., 2015). Further analysis and comparison of the response patterns of tobacco *MAX* family members to other stresses such as light, pests and diseases, and hormones such as brassinolide is still needed.

## Conclusion

This study presents the phylogeny, gene structure, expression levels, and cis-acting elements of the *MAX* gene family in representative solanaceous crops using bioinformatics approaches. There were 74 non-redundant MAX family proteins identified in tobacco, tomato, potato, petunia, and pepper. These MAX genes showed tissue-specific expression. Furthermore, cis-acting element analysis showed that the identified *MAX* genes contained cis-elements that are predicted to respond to hormones, stress, light, and shoot development. MAX1–MAX4 were all upregulated in respond response to GR24 and topping treatments. Our identification and analysis of the *MAX* gene family in solanaceous crops provides insights for further research into the role of tobacco *MAX* genes in axillary development and stress resistance.

## Supplemental Information

10.7717/peerj.14669/supp-1 Supplemental Information 1 Genome-wide identification of MAXs genes for SLs synthesis/signaling in solanaceous plants and analysis of their potential functions in tobacco.

10.7717/peerj.14669/supp-2 Supplemental Information 2 Cis-acting elements.

10.7717/peerj.14669/supp-3 Supplemental Information 3 The original experimental data of fluorescence quantitative PCR.

10.7717/peerj.14669/supp-4 Supplemental Information 4 The representative sequence diagrams of motif 1 to motif 10.

10.7717/peerj.14669/supp-5 Supplemental Information 5 The protein sequences of 89 MAX family members (including in Arabidopsis and rice).

## Additional Information and Declarations

### Data Availability

The following information was supplied regarding data availability: The raw data is available in the Supplemental Files.
