# Supplementary figures and images for: Genome-wide identification of MAXs genes for strigolactones synthesis/signaling in solanaceous plants and analysis of their potential functions in tobacco
Source: PeerJ. 2023 Jan 12;11:e14669. doi: 10.7717/peerj.14669 (PMC9840856; doi:10.7717/peerj.14669)

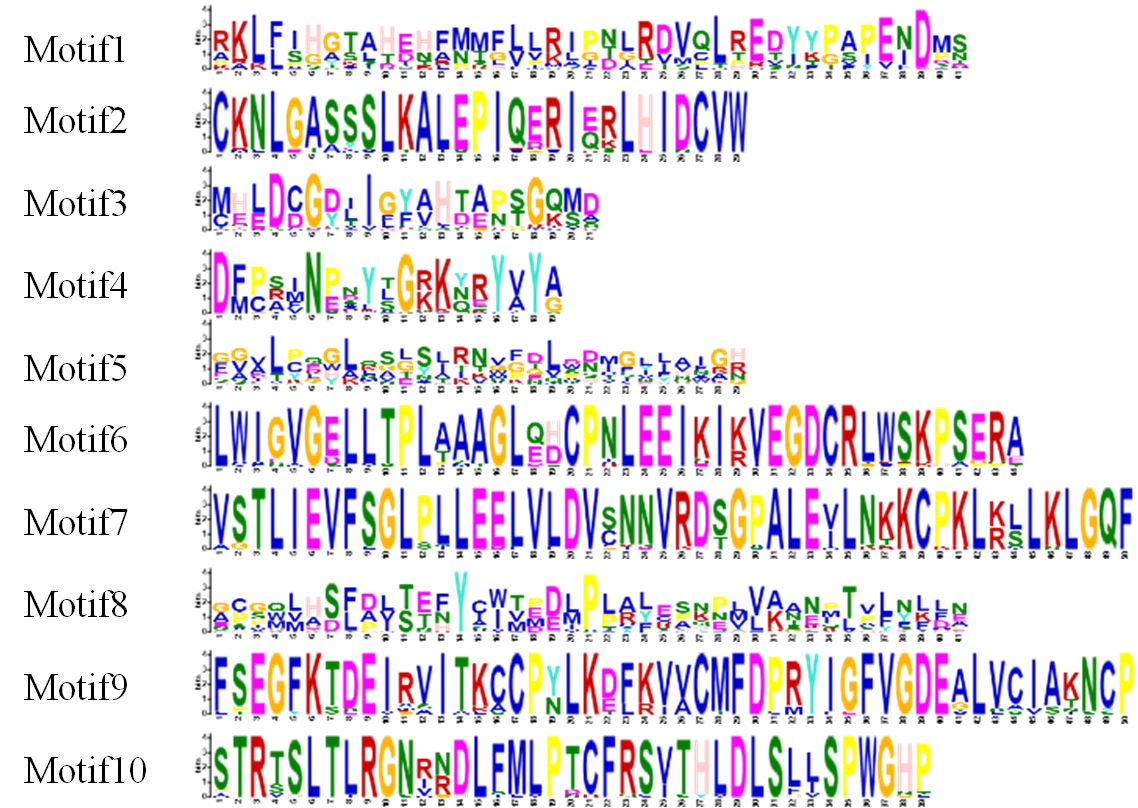

Supplement: Supplemental Information 4 [file peerj-11-14669-s004.png]
